# The relationship between levels of Alexithymia and communication skills of nursing students

**DOI:** 10.12669/pjms.35.2.604

**Published:** 2019

**Authors:** Behire Sancar, Demet Aktas

**Affiliations:** 1 *Behire Sancar, PhD. Assistant Professor, Department of Nursing and Health Services, Toros University, School of Health Sciences, Mersin, Turkey*; 2 *Demet Aktas, PhD. Assistant Professor, Department of Nursing and Health Services, Toros University, School of Health Sciences, Mersin, Turkey*

**Keywords:** Alexithymia, Communication skill, Nursing student

## Abstract

**Background & Objective:**

Effective communication in the nursing profession is not just a personal skill but is accepted as a learned and gained technique in the instructional process. It is possible for nurses to professionally provide effective and quality service with the establishment, development, and transfer to emotion of effective communication with people. The purpose of this study was to investigate the relationship between levels of alexithymia and communication skills of nursing students.

**Methods:**

This cross-sectional study was conducted among students attending the School of Nursing in a university in Turkey in the 2017-2018 Spring semester. A total of 634 nursing students participated in the study. The data in the study were collected with the “Student Introduction Form”, the “Toronto Alexithymia Scale (TAS)” and the “Communication Skills Scale (CSS)”. Means, standard deviations, t-test, ANOVA and Pearson correlation analysis were used for the analysis.

**Results:**

The mean TAS scores of the nursing students were found to be 56.31±8.82, and the students had “moderate alexithymia” based on the average scores of the scale. The mean CSS scores of the nursing students in the study was found to be 91.16±12.99, and the students had “Good level of communication” based on the average scores of the scale. In our study, a negative and moderate correlation between the levels of alexithymia of nursing students and their communication skills was detected (p: 0.001).

**Conclusion:**

It was found that as the levels of alexithymia of nursing student increased, their communication skills decreased.

## INTRODUCTION

Effective communication in the nursing profession is not just a personal skill but is accepted as a learned and gained technique in the instructional process.^1^,^2^ It is possible for nurses to professionally provide effective, constant and quality service through establishing, developing and transferring emotions of effective communication with people. In terms of effective communication, it is important for nurses who are in an occupational state of intense communication with ill individuals at every age and socioeconomic and cultural level to trust and empathize with the individuals with whom they establish communication in the initiation, continuation, and conclusion of communication.^3^,^4^ Since effective communication between nurses and patients provides positive contributions to the process of the patient dealing with stress, producing solutions to problems, acclimating to the disease and recovering, increases the quality of patient care, patient satisfaction, and job satisfaction.^5^,^6^

In the occupation of professional nursing, providing care services to bring the health statuses of individuals in society up to the highest level, there is need for educators and professionals who are confident in themselves, cooperative, aware of the problems of themselves and others, and can establish effective communication.^2^,^5^,^7^ Educators who are good role models will lead students to establish positive social relationships with their colleagues and environments, additionally, they will be influential in understanding their behaviors, and in developing creative alternatives in predicting and solving problems that may develop.^8^

In the process of effective communication, a nurse must be aware of and fulfill the functions of recognizing and expressing their own and others’ emotions and empathizing.^9^ Any kind of fault that occurs in the fulfillment of these functions by nurses will cause disconnection on communication and reflect negatively on the quality of care of the patient. The issue in the ability to recognize and express feelings is generally defined as alexithymia.^10^,^11^ Previously conducted studies report that alexithymia occurs at a rate of 10% in healthy individuals.^12^-^15^ Alexithymia negatively affects the ability of individuals to empathize and makes the experience of communication problems inescapable.^10^,^12^,^15^

In the process of quality and effective communication, individuals’ ability of understanding, expressing, and being aware of emotions carries great importance. This is why the specification of the levels of alexithymia, the examination of whether there is a relationship between levels of alexithymia and communication skills, and the development of activity programs directed at increasing emotional awareness are important for students in the nursing education process. No study was encountered in the literature in which the relationship between the level of alexithymia in the field of nursing and communication skills were studied.

The purpose of this study was to investigate the relationship between their levels of alexithymia of and communication skills of nursing students.

## METHODS

This is a cross-sectional study.conducted on students attending the School of Nursing in a university in Turkey in the 2017-2018 Spring semester. The study’s population of the nursing students attending the School of Nursing was formed and a sample selection was made. The total number of the nursing students attending the School of Nursing was 811 of which 634 (79.25%) agreed to participate in this study. The selection criteria in the study were specified as all volunteer nursing students over the age of 18.

Data in the study were collected using and the “Student Introduction Form (SIF)”, the “Toronto Alexithymia Scale (TAS)” and the “Communication Skills Scale (CSS)”. The “SIF” contains some socio-demographic characteristics of students (age, gender etc.) and, it was structured by the researchers after investigation of previous studies in the literature.^3^-^6^ The second part comprised of “CSS” developed by Korkuk Owen and Bugay in 2014 was used to measure communication skills of university students.^16^ The CSS is a 5-point Likert-type scale consisting of 10 items and 4 sub-dimensions. The subdimensions of the scale are as follows: Communications Principles and Fundamental Skills (CPFS), Self-Expression (SE), Effective Listening and Nonverbal Communication (ELNC), Willingness to Establish Communication (WEC). The points obtained from the scale being high indicate that the communication skills of the individuals are developed. In this scale, the lowest and highest scores are 25 and 125, respectively. The Cronbach alpha value for the CSS is 0.81.^16^ The “TAS” developed by Taylor et al in 1985 was used to determine the alexithymia-related characteristics of individuals. The TAS is a 5-point Likert-type scale consisting of 26 items and 4 sub-dimensions.^17^ However, the scale was later minimized to 20 items and 3 sub-dimensions. The Cronbach alpha value for the TAS is 0.78.^18^ There are seven items in the Difficulty Recognizing Emotions (DRE), five items in the Difficulty Expressing Emotions (DEE), and eight items in the Extroverted Thought Structure (ETS), which are all sub-dimensions of the scale.^18^ The lowest score that could be received from the TAS was “20”, and the highest was “100”. Persons who received “51 points and below” from the scale do not have alexithymia, but persons who receive “52-61” points are defined as having moderate alexithymia, and persons who receive “62 points and above” are defined as having “alexithymia”.^10^ The study was carried out by the researchers at the beginning of the class hour with permission of the instructors of the related departments. The data gathering covered approximately 20 minutes.

### Ethical considerations

Institutional ethics committee approved the study, and written informed consents were obtained from the students who agreed to participate in the study.

### Data analysis

The SPSS 22.0 software (SPSS Inc., Chicago, IL, USA) program was used in the analysis of the data. Mean, t-test, ANOVA and Pearson correlation analysis were used for the analysis.

## RESULTS

The study was completed with the participation of 634 nursing students. The mean age of the students was 20.8±2.32 and the mean academic grade point average (GPA) was 2.81±0.42. Of the students, 39.0% were second-year students. It was reported that 78.7% of the students who participated in the study were 21 years of age and younger, that 84.2% were female, that the family type of 82.2% was a nuclear family, that the income level of 76.9% was moderate, and that 61.8% had a “good” level of interpersonal communication. Of the students, 61.5% lived in the province and 48.9% lived with their families. It was determined that the educational levels of the 74.8% of the mothers and 44.3% of the fathers of the students were at or below the level of secondary education. The mean TAS scores of the nursing students were found to be 56.31±8.82, and the students had “moderate alexithymia” based on the average scores of the scale. It was reported that the highest average scores that students received from the TAS subdimensions were in the subdimensions of ETS (25.67±4.34), DRE (19.08±5.64), and DEE (14.33±2.65), respectively.

The total mean scores for the TAS and its subdimensions (ETS, DRE, DEE) were found to be significantly lower for female students than for male students, for students whose GPA is 2.51 and above than for students whose GPA is 2.50 and below, and for students whose interpersonal communications level is “good” than for students whose interpersonal communications level is “moderate” and “poor”; and the difference between the groups is statistically significant (Table-I; p<0.05).

**Table-I:** TAS and sub-dimension outcomes comparisons of some characteristics of students (n: 634).

Characteristics	n (%)	DRE	DEE	ETS	Total TAS
***Gender***					
Female	534 (84,2)	18.77 ± 5.38	14.28 ± 2.57	25.58 ± 3.74	55.91 ± 8.09
Male	99 (15,8)	20.73 ± 6.67	15.62 ± 3.04	26.14 ± 6.74	58.46 ± 11.85
p		0.006	0.014	0.023	0.003
***GPA (four ’system)***					
≤ 2,50	150 (23,6)	20.91 ± 6.16	16.66 ± 2,62	27.45 ± 5,80	58.14 ± 10.53
≥ 2,51	484 (76,4)	18.51 ± 5.36	14.22 ± 2,65	25.72 ± 3,79	55.72 ± 8.15
p		0.001	0.025	0.004	0.004
***Level of interpersonal communication***					
Good^a^	392 (61.8)	18.03 ± 5.60	14.11 ± 2.78	25.88 ± 4.81	55.29 ± 9.55
Moderate^b^	228 (35.9)	20.49 ± 5.09	15.66 ± 2.39	25.34 ± 3.41	57.71 ± 7.01
Weak^c^	14 (2.3)	25.71 ± 6.15	16.85 ± 2.24	24.85 ± 3.65	62.21 ± 8.90
p (a-b), (a-c), (b-c)		0.001	0.003	0.004	0.001

The mean CSS scores of the nursing students in the present study were found to be 91.16±12.99. In the present study, it was found that the lowest average scores that the students received from the CSS and its subdimensions were in the SE (15.17±2.84), WEC (18.47±3.15), ELNC (23.15±3.75) and CPFS (38.35±5.76) subdimensions, respectively.

In our study, the total mean scores for the CSS and its subdimensions (CPFS, SE, ELNC, WEC) were found to be significantly higher for female students than for male students, for students whose GPA is 2.51 and above than for students whose GPA is 2.50 and below, and for students whose interpersonal communications level is “good” than for students whose interpersonal communications level is “moderate” and “weak”; and the difference between the groups was found statistically significant (Table-II; p<0.05).

**Table-II:** CSS and sub-dimension outcomes comparisons of some characteristics of students (n: 634).

Characteristic	n (%)	CPFS	SE	ELNC	WEC	Total CSS
***Gender***						
Female	534 (84.2)	38.66 ± 5.61	16.25 ± 2.75	23.35 ± 3.58	19.49 ± 3.09	91.77 ± 12.54
Male	99 (15.8)	36.68 ± 6.31	14.74 ± 3.26	22.15 ± 4.42	18.43 ± 3.47	88.01 ± 14.95
p		0.007	0.019	0.002	0.003	0.018
***GPA (four ’system)***						
≤ 2.50	150 (23.6)	36.91±6.03	14.44±3.08	22.52±4.16	17.97±3.27	87.93±13.99
≥ 2.51	484 (76.4)	38.79±5.63	16.39±2.73	24.35±3.60	19.68±3.10	92.15±12.54
p		0.001	0.001	0.019	0.027	0.001
***Level of interpersonal communication***						
Good^a^	392 (61.8)	39.08±5.91	15.83±2.72	23.73±3.80	19.18±3.12	93.75±13.24
Moderate^b^	228 (35.9)	37.37±5.22	14.17±2.65	22.20±3.50	17.34±2.85	87.24±11.44
Worse^c^	14 (2.3)	33.78±6.06	12.78±3.14	22.50±3.39	17.28±2.99	82.64±10.26
p (a-b),(a-c), (b-c)		0.001	0.001	0.001	0.001	0.001

In our study no statistically significant differences were found between nursing students’ age, class, mother-father educational level, income level, their place of residence, the people they live with, or family type and, the mean scores of the TAS and the CSS (p>0.05).

In addition, in our study, there was found to be a negative, moderate correlation between the mean scores for the TAS and its subdimensions (DRE, DEE) of and the mean scores of the CSS and its subdimensions (CPFS, SF, ELNC, WEC) of the nursing students. This correlation was found to be statistically significant (Table-III; p: 0.001).

**Table-III:** The correlation between the mean scores of TAS and CSS of students.

Scale and sub-dimension	CPFS	SE	ELNC	WEC	Total CSS
Total TAS	r: - 0.527	r: - 0.593	r: - 0.485	r: - 0.436	r: - 0.408
	p: 0.001	p: 0.001	p: 0.001	p: 0.001	p: 0.001
DRE	r: - 0.357	r: - 0.398	r: - 0.428	r: - 0.364	r: - 0.323
	p: 0.001	p: 0.001	p: 0.001	p: 0.001	p: 0.001
DEE	r: - 0.657	r: - 0. 589	r: - 0.673	r: - 0.478	r: - 0.571
	p: 0.001	p: 0.001	p: 0.001	p: 0.001	p: 0.001
ETS	r: 0.200	r: 0.114	r: 0.148	r: 0.111	r: 0.085
	p: 0.217	p: 0.829	p: 0.701	p: 0.587	p: 0.073

## DISCUSSION

Humans are social beings.^9^ In the process of effective communication the recognition, awareness, and expression of human emotions and the understanding of the emotions of the opposing individual carry distinct importance and share similarities with effective communication in the nursing profession based on professional care services.^7^,^10^,^19^ The nurse dealing with his or her patient as a whole and understanding them, identifying their needs, and being able to obtain results that are satisfactory of the care services can be provided with communication skills being strong. In a study conducted by Kumcağız et al,^2^ it was specified that especially nursing students needed to gain some characteristics such as being able to establish effective communication, being able to express their emotions or thoughts, and being able to distinguish their physical senses from their emotions.^2^ Our study found that nursing students had “moderate alexithymia” and that the students struggled moderately in recognizing and expressing their emotions and in their extroverted thoughts. In another study, it was reported that nurses have moderate alexithymia and experience difficulty in communication with patients.^10^ It is reported that the difficulties nursing students experience with regard to emotional awareness leads to them being unable to understand the underlying meaning of patients’ verbal-nonverbal thoughts and feelings and causes difficulties in communication.^10^,^20^ The results of the present study lead us to think that nursing students who are unable to comfortably reflect their emotions on their environment may experience problems in terms of effective communication.

Our study results showed that the mean scores for total alexithymia and its subdimensions in terms of the gender variable were significantly lower for female students than for male students. Previously conducted studies emphasized that the emotional brains of women developed better relative to males and that the levels of alexithymia were lower relative to males.^15^,^17^

Our study found that the mean scores of total alexithymia and its subdimensions in terms of the academic success variable were lower for students with high GPA than students with low GPA. It was reported that academic success in students nourished and strengthened interpersonal communication and prevented the development emotions such as emotional difficulty, and prevented the development of alexithymia. Therefore, a close relationship is found between academic success and alexithymia.^21^ The increase of level of GPA of the students increases self-confidence and the power to express themselves and allows for the strengthening of communication skills by reducing the characteristics of alexithymia.

In the current study, it was found that the total mean scores of alexithymia and its subdimensions in terms of the interpersonal communication level variable were lower for students with “good” interpersonal communication levels than for students with “moderate” and “poor” interpersonal communication levels. Sasioglu et al and Aaron et al reported in the studies they conducted that alexithymia deteriorates positive interpersonal relationships and makes individuals aggressive, conflictive, introverted in understanding and expressing emotions, and indifferent.^19^,^21^ These results show that, as the average alexithymia score increases, difficulty in interpersonal relationships can be experienced, and the results of our study are consistent with the literature.

It was found in our study that the communication skills of the nursing students were at a “good” based on the mean scores of the CSS and its subdimensions (communication principles and fundamental skills, self-expression, effective listening and nonverbal communication, willingness to establish communication). Previously conducted studies reported that the communications skills of nursing students were high.^5^,^8^ Nurses’ usage effective communications skills in the working field is important in terms of preventing conflicts within the team, uncertainty in interprofessional roles, and power struggles. The effective communication of nurses with the patients to whom they provide care is also guiding in terms of ensuring a therapeutic relationship, facilitating their skills of empathy, being able to comfortably express their emotions, and developing problem-solving skills.^2^

In our study, it was found that female students, students with high academic success and high level of interpersonal communication were higher good of communication skills (p<0.05). Previously conducted studies reported that the communication skills of female students were at a significantly higher degree than that of male students.^3^,^4^ Similarly, it was reported that the communication and empathizing skills and self-confidence of students with good academic success and interpersonal relations were high.^9^

We also observed a negative and moderate correlation between the mean scores of the TAS and its subdimensions (DRE, DEE) and the mean scores of the CSS and its subdimensions (CPFS, SE, ELNC, WEC) of nursing students (p<0.05). In previous studies it was determined that individuals with alexithymia take measures to prevent themselves from leading a superficial life far from the inner world that contains no emotion and progresses like a robot and from not going deeper into the problems they face but superficially resolving them and preventing them from reemerging.^10^,^12^,^20^ This situation leads to individuals who are unable to comfortably explain their emotions to their surroundings struggling in the communication process.

## CONCLUSION

Nursing students have moderate alexithymia but their communication skills were at a “good level”. Interpersonal communications level is “moderate” and “poor” meaning as the levels of alexithymia increase, their communication skills decrease. In nursing education, the teaching of methods that increase the emotional awareness of students and that aid their effective communication and the guidance to laboratory and clinical practices to teach them to recognize emotions and communication skills are recommended.

### Author`s Contribution

**BS and DA:** Study design, data collection-analysis, and manuscript preparation.
